# Enhanced photoluminescence of multilayer Ge quantum dots on Si(001) substrates by increased overgrowth temperature

**DOI:** 10.1186/1556-276X-7-383

**Published:** 2012-07-11

**Authors:** Zhi Liu, Buwen Cheng, Weixuan Hu, Shaojian Su, Chuanbo Li, Qiming Wang

**Affiliations:** 1 State Key Laboratory on Integrated Optoelectronics, Institute of Semiconductors, Chinese Academy of Sciences, QingHua East Road, Haidian District, Beijing, 100083, People’s Republic of China

**Keywords:** Ge quantum dots, Photoluminescence, Ostwald ripening, Overgrowth temperature

## Abstract

Four-bilayer Ge quantum dots (QDs) with Si spacers were grown on Si(001) substrates by ultrahigh vacuum chemical vapor deposition. In three samples, all Ge QDs were grown at 520 °C, while Si spacers were grown at various temperatures (520 °C, 550 °C, and 580 °C). Enhancement and redshift of room temperature photoluminescence (PL) were observed from the samples in which Si spacers were grown at a higher temperature. The enhancement of PL is explained by higher effective electrons capturing in the larger size Ge QDs. Quantum confinement of the Ge QDs is responsible for the redshift of PL spectra. The Ge QDs’ size and content were investigated by atomic force microscopy and Raman scattering measurements.

## Background

Si-based light emitter is one of the most important components for Si-based photonic integration. Although many progresses have been made for silicon-based light emitter in recent years [1-4], it is still a big challenge to overcome the inefficient band-to-band radiative recombination of silicon. With large band offset and strong quantum confinement, the self-assembled QDs are promising structure to enhance the optical characteristics [5]. In the past two decades, the self-assembled Ge QDs on Si substrates, which are compatible with complementary metal-oxide semiconductor processes, have been widely studied for Si-based optoelectronic device applications [6,7]. Unfortunately, the Ge QDs on Si can only provide a good confinement for the holes, which is hard to capture the electrons. Lacking of electrons for radiative recombination in Ge QDs limits its emission efficiency. A lot of efforts had been made to investigate luminescence of Ge QDs/Si(001) multilayer structure [8-10]. However, the radiative recombination in Ge QDs is still weak, even observed at low temperature [8,9,11]. How to increase the radiative recombination of Ge QDs is still a problem. Usually, carrier collection is a size-dependent behavior [12]. Therefore, increasing Ge QDs’ probability of capturing the electrons by increasing Ge QDs’ size is a feasible way to improve the Ge QDs’ emission performance. However, many studies concentrated on lower temperature grown small-size Ge QDs [6,13], which have stronger quantum confinement and lower Si-Ge interdiffusion.

In this work, we balance the advantages of small-size Ge QDs (strong quantum confinement and low Si-Ge interdiffusion) and the advantages of large-size Ge QDs (high electron capture probability). Ostwald ripening of Ge QDs induced by higher Si spaces’ overgrowth temperature was used to obtain large-size Ge QDs. The PL spectra obtained from the sample in which Si spacers were grown at higher temperature show a significant signal enhancement.

## Methods

Three samples were grown by cold-wall UHV-CVD on Si(001) substrates with a resistivity of 2 to approximately 4 Ω cm, using pure disilane (Si_2_H_6_) and germane (GeH_4_). The Si substrates were first cleaned using an *ex situ* improved RCA wet-chemical cleaning recipe and then loaded into the pretreatment chamber. Before growing, the substrate was degassed at 300 °C for several hours in the pretreatment chamber and then was heated up to 920 °C for 5 min in the growth chamber with a background pressure lower than 1 × 10^−7^ Pa to deoxidize. Next, a 15-nm-thick Si layer was grown at 520 °C (sample A), 550 °C (sample B), and 580 °C (sample C), respectively, to obtain a flat starting surface. After a 240-s growth interruption to change the growth temperature, 5 monolayers (ML, 1 ML = 6.27 × 10^14^ Ge atom cm^−2^) of Ge was deposited at 520 °C with a rate of 0.04 Å/s. After a second growth interruption to change the growth temperature, the next three bilayer was grown in the same way. In order to study the morphology of Ge QDs, the top Ge QDs are not covered with Si cap. For all samples, the thickness of Ge QDs and Si spacer was 5 ML and 15 nm, respectively. All Si spacers were grown below 600 °C to prevent the Si-Ge interdiffusion [14,15]. The reflection high-energy electron diffraction system was used to *in situ* monitor the growth of Ge QDs and Si spacers. The surface morphology of the samples was examined by the AFM, which was performed in contact mode. Scanning transmission electron microscopy (STEM) was used to study the Ge QDs growth behavior in the multilayer structure. PL (Raman) measurements were performed with LabRam HR 800 Raman instrumentation (HORIBA Jobin Yvon Inc., Paris, France) at room temperature, using a 488-nm-line Ar^+^ laser with the laser power of 15 (5) mW and an InGaAs photodetector within 1,150 to 1,600-nm range.

## Results and discussion

**Figure 1:**
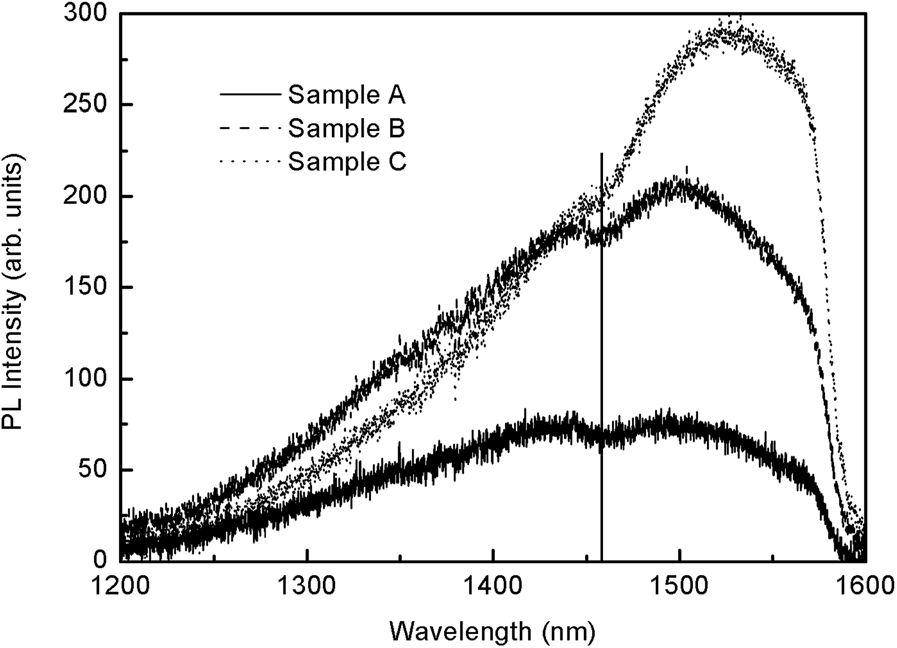
**Room temperature PL spectra of four-bilayer samples.** With the Si spacer overgrowth at 520 °C (sample A), 550 °C (sample B), and 580 °C (sample C). The PL decrease around 1,460 nm induce by the color filter of instrumentation is marked by a line.

**Figure 2:**
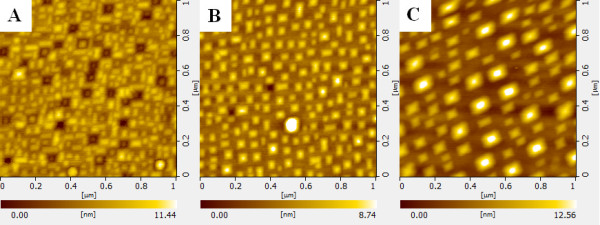
**AFM images (1 μm × 1 μm) of four-bilayer samples.** With the Si spacer overgrowth at 520 °C (sample **A**), 550 °C (sample **B**), and 580 °C (sample **C**).

Room temperature PL results of the three samples are depicted in Figure 1. Appreciable PL intensity enhancement was observed for sample B (the middle curve in Figure 1) and sample C (the top curve in Figure 1) in which Si spacers were grown at 550 °C and 580 °C, respectively. The different PL intensity is induced by various Ge QDs’ size. Figure 2 shows the 1 μm × 1 μm AFM images of the surface morphology of three samples. The Ge QDs’ size differences induced by Ostwald ripening [16,17] among three samples are observed cleanly. The morphology of sample A dominates by the small-size hut shape of Ge QDs, which has the highest density of QDs in all samples. However, sample A’s PL intensity is the lowest. Samples B and C have larger Ge QDs and lower density of Ge QDs; however, the PL intensity shows a significant enhancement. More details of Ge QDs AFM statistic analysis is shown in Table 1.

**Table 1:** Summary of the AFM statistic analysis of samples

**Sample**	**Width (nm)**	**Height (nm)**	**Density (10**^**10**^ **m**^**−2**^**)**
A	32 ± 5	2.5 ± 0.3	3.8
B	50 ± 8	4.4 ± 0.5	2.4
C	92 ± 10	8.1 ± 1	0.5
	30 to 60	2.4 to 5	0.6

According to Fermi’s age equation [18] and Ge QDs’ sizes from AFM, we estimate the capture probability for electrons of Ge QDs in different samples. The probability in sample C is about 1.6 and 1.2 times higher than that of sample A and B, respectively. Further, the nonradiative recombination channels related to point defects that form at low growth temperature can decrease the PL intensity [11,19]. The growth process of Ge QDs in which Si spacers were grown at higher temperature are similar to cyclic annealing. It may decrease the point defects and improve the crystal quality [20].

**Figure 3:**
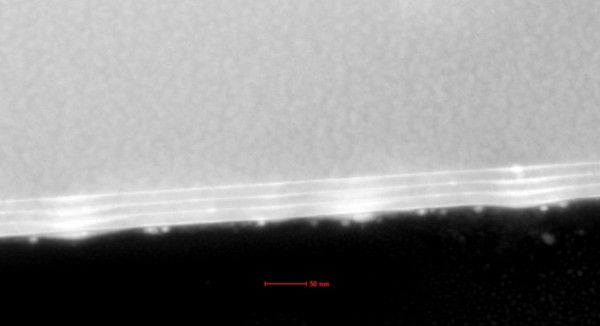
**STEM image of sample C with the Si spacer overgrowth at 580 °C.** The length scale is 50 nm.

Figure 3 is the typical STEM image of sample C. It is found that the Ge QDs have a good vertical coupling in the multilayer structure. Top Ge QDs have a little larger size than the buried ones. Overall, the Ge QDs’ size obtained from STEM image is in agreement with the results from AFM.

The room temperature PL spectra from all samples consisted of a broad peak and a shortwave tail. It is noteworthy that the PL decreases around 1,460 nm, which is induce by the color filter of instrumentation. The broad peaks are attributed to the emission of Ge QDs. The shortwave tail is attributed to the disunity size distribution of Ge QDs [19] and Si-Ge interdiffusion [9,10]. Although the sample A’s PL spectra is too broad to distinguish the accurate PL peaks, a remarkable redshift of PL spectra can be observed among three samples. The PL peaks of samples B and C are around 1,500 and 1,530 nm, respectively. The energy of the redshift between samples B and C is 16 meV. This peaks’ shift in PL spectra is induced by Si-Ge interdiffusion and quantum confinement effect. PL energy of Ge QDs is given by the following:

(1) $$E_{\text{PL}}=E_{\text{gap,Si}}-\Delta E_{\text{v}}+\Delta E_{\left(nmk\right)}\text{,}$$

where *E*_gap,Si_ is the bulk Si band gap; *ΔE*_v_, the valence band offset of Ge on Si which depended on the content of the Ge QDs; and *ΔE*_(*nmk*)_, the confinement energy shift of the Ge QDs. Therefore, we calculate the average content and the energy shift of the Ge QDs in the samples.

**Figure 4:**
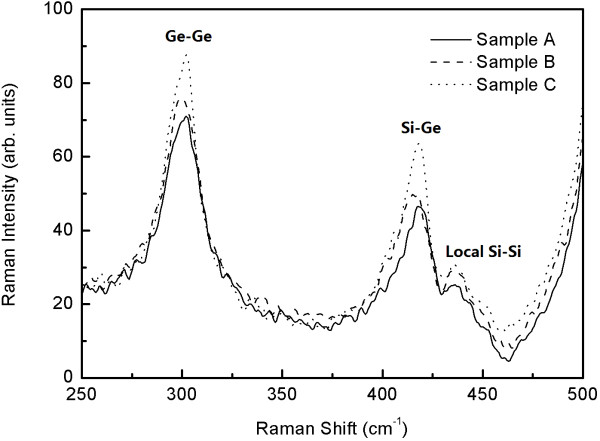
**Raman spectra of three samples.** With the Si spacer overgrowth at 520 °C (sample A), 550 °C (sample B), and 580 °C (sample C).

Figure 4 shows the Raman spectra of the three samples. The Raman peaks related to scattering at the Ge-Ge vibrations (about 300 cm^−1^) and the Si-Ge vibrations (approximately 415 cm^−1^) accompanying with a small local Si-Si vibrations (about 430 cm^−1^) can be seen in the spectra. Raman intensity enhancement and a little shift of Si-Ge peak is observed. The Raman intensity enhancement indicates that the growth process of Ge QDs in which Si spacers were grown at higher temperature benefits its crystal quality. The content of Ge QDs can be calculated from the ratio between the integrated intensities of the Raman peaks corresponding to the Ge-Ge and Ge-Si bonds, *I*_Ge-Ge_ and *I*_Si-Ge_[21,22]:

(2) $$x=\frac{2I_{\text{Ge-Ge}}/I_{\text{Ge-Si}}}{\alpha +2I_{\text{Ge-Ge}}/I_{\text{Ge-Si}}}$$

The parameter *α* is a constant which depends on the experimental conditions. We obtained *α* from the Raman spectra of many samples with various compositions of Ge-rich Ge_*x*_Si_1 −__*x*_ layers, in which the Ge contents are calculated from X-ray diffraction measurements. In this way, we determined the coefficient *α* = 1 for our experimental conditions. The average Ge contents in Ge QDs of three samples are 71%, 70%, and 70% corresponding to samples A, B, and C, respectively. The difference of Ge contents in Ge QDs induces the shift of Si-Ge peak. The average Ge contents of Ge QDs were almost similar; therefore, the Si-Ge interdiffusion is not the reason for the redshift of PL spectra. Further, we calculate the energy shift in QDs by the well-known expression:

(3) $$\Delta E_{\left(nmk\right)}=\frac{\pi h^{2}}{2m^{*}}\left(\frac{n^{2}}{h^{2}}+\frac{m^{2}}{w^{2}}+\frac{k^{2}}{w^{2}}\right)$$

where *n*, *m*, *k* = 1, 2,… are the quantum numbers for coordinates *z*, *x* and *y*, respectively. m* = 0.28 *m*_0_ is the effective mass of heavy holes of Ge (*m*_0_ is the mass of a free electron). According to Equation 3, the value of *ΔE*_111_ = 22 and 7 meV corresponding to samples B and C. In this way, the redshift of PL is 15 meV. It can be seen that the experimental data is in good agreement with the results of calculations based on the model used here. Therefore, the reason of redshift of PL spectra is the quantum confinement in Ge QDs.

Besides, we notice some interesting square nanopits’ morphology, with a depth of about 7 nm and contains small Ge QDs which are formed in the Si spacer layer (Figure 2A). Similar morphology was described in the literature [23]. They believe that Si spacer grown at low temperature has higher strain. The Si atoms of high-strained Si mounds formed over the Ge QDs migrate to the surrounding area responsible for the nanopits.

## Conclusions

In summary, we obtain large-size Ge QDs below 600 °C by Ostwald ripening of Ge QDs which is induced by higher Si spaces’ overgrowth temperature. Enhancement and redshift of room temperature PL were observed from the sample which have larger size Ge QDs. Large-size Ge QDs have more probability to capture the electrons for radiative recombination which is responsible for the PL intensity enhancement. Si spacers grown at higher temperature can improve the crystal quality. The redshift of PL peaks is attributed to the quantum confinement of Ge QDs.

## Competing interests

The authors declare that they have no competing interests.

## Authors’ contributions

ZL and BC designed the study and carried out the experiments. ZL, WH, and SS performed treatment of experimental data and calculations. ZL, BC, CL, and QW took part in the discussions of the results and prepared the manuscript initially. All authors read and approved the final manuscript.
